# First report of perioperative iptacopan interruption in paroxysmal nocturnal hemoglobinuria without breakthrough hemolysis

**DOI:** 10.1016/j.htct.2025.106235

**Published:** 2025-12-13

**Authors:** Katsuhiro Tokuda, Tatsuki Morioka, Shoya Arai, Takuji Matsuo, Kensuke Matsumoto, Ryosuke Shirasaki, Jun Ooi, Haruko Tashiro

**Affiliations:** a Department of Hematology/Oncology, Teikyo University School of Medicine, 2-11-1, Kaga, Itabashi-ku, Tokyo 173-8606, Japan; b Department of Clinical Laboratory Science, Teikyo University, 2-11-1, Kaga, Itabashi-ku, Tokyo 173-8606, Japan

## Introduction

Paroxysmal nocturnal hemoglobinuria (PNH) is a rare clonal hematopoietic stem cell disorder characterized by complement-mediated intravascular hemolysis, thrombosis, and marrow failure. Iptacopan, an oral selective factor B inhibitor, has emerged as a novel therapeutic agent that effectively controls hemolysis and reduces transfusion burden by targeting the alternative complement pathway [1]. Its oral administration provides convenience but raises concerns over adherence and peri‑interruption risk, especially in the surgical setting where oral intake may be restricted. Perioperative management of iptacopan remains undocumented, and no case reports have described the safety of temporary drug interruption in surgical settings.

We herein report, to our knowledge, the first case of successful laparoscopic cholecystectomy following brief preoperative interruption of iptacopan without breakthrough hemolysis (BTH), highlighting a practical approach to perioperative management of this novel oral complement inhibitor.

## Case report

A 57-year-old woman with a history of depression and ovarian cysts presented in September 2022 with fatigue, anemia, and progressive thrombocytopenia. Initial laboratory testing showed hemoglobin 5.6 g/dL with macrocytosis (MCV: 115.9 fL), platelet count 32 × 10^9^/µL, and white blood cell count 4.7 × 10^9^/L without differential abnormality. Lactate dehydrogenase (LDH) was slightly elevated (241 IU/L) and haptoglobin was at an undetectable level (<2 mg/dL). Flow cytometry identified 5.9 % of CD55/CD59-deficient erythrocytes, which later increased to 31.8 %. Bone marrow examination showed mild dyserythropoiesis and slightly reduced megakaryocytes, no blast excess, and a normal karyotype. A diagnosis of PNH with possible coexisting myelodysplastic syndrome was made.

Immunosuppressive therapy with cyclosporine was ineffective. In January 2024, ravulizumab was initiated after a meningococcus vaccination (Menactra®), reducing the red blood cell (RBC) transfusions from 4 to 2 units per month but without transfusion independence. In June 2024, danicopan (150 mg three times daily, later increased to 200 mg) was added due to persistent anemia but this remained insufficient. During this period, the patient developed cholecystitis and experienced BTH.

In December 2024, therapy was switched from ravulizumab and danicopan to iptacopan (200 mg twice daily) following pneumococcal (Pneumovax®23) and haemophilus influenzae type b (ActHIB®) vaccinations, with the first dose given six weeks after the last ravulizumab infusion. Hemoglobin stabilized, and transfusions were no longer required.

**Figure 1:**
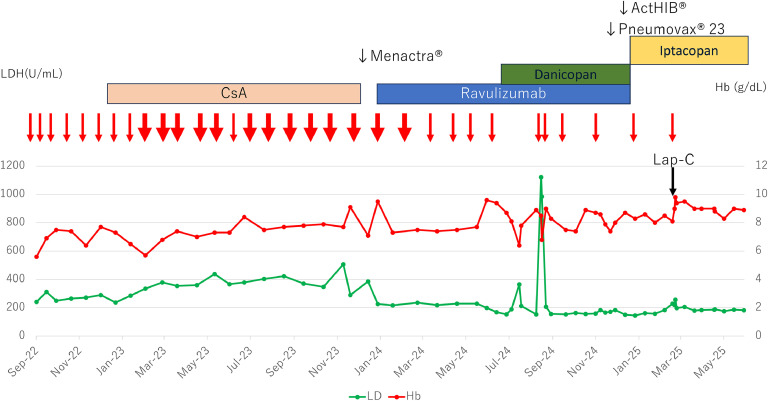
Clinical course of the patient. LDH: lactate dehydrogenase; CsA: Cyclosporine A; Hb: hemoglobin; Lap-C: laparoscopic cholecystectomy thin arrow: 2 units red blood cell transfusion; thick arrow: 4 units red blood cell transfusion.

Elective laparoscopic cholecystectomy was scheduled for March 2025. On admission, the hemoglobin level was 8.1 g/dL, which would not typically require transfusion. However, considering the upcoming surgery, two units of RBC were administered. Considering the pharmacokinetics of iptacopan (T_max ≈ 2 h, half-life 18–25 h) [2], a one-day interruption (two missed doses) was planned. The final preoperative dose was taken at 7:00 pm on the day before surgery; both doses on the surgery day were withheld. Eculizumab was kept on standby in case of prolonged oral intake restriction or BTH. Oral iptacopan was resumed at 7:00 am on postoperative Day 1. Blood tests at 5:00 am that day showed stable hemoglobin and a mild LDH increase (256 IU/L), which normalized by postoperative Day 3. No hemoglobinuria, vital sign changes, or thrombotic events occurred. The patient recovered uneventfully and has remained on iptacopan, with sustained transfusion independence and improvement in fatigue. The perioperative laboratory data, with stable hemoglobin, LDH and indirect-bilirubin levels, are summarized in Table 1. The clinical course from diagnosis to the present is illustrated in Figure 1.

**Table 1:** Perioperative laboratory data.

	Day −3	Day 0	POD 1	POD 3	POD 14
Hb (g/dL)	8.1*	9.0	9.8	9.4	9.5
LDH (IU/L)	229	220	256	197	205
ID-Bil (mg/dL)	0.64	0.77	0.90	0.69	0.68
CRP (mg/dL)	0.18	0.12	2.31	4.08	1.59

POD: Post-operative day; Hb: hemoglobin; LDH: lactate dehydrogenase; ID-Bil: indirect bilirubin; CRP: C-reactive protein.

⁎ Preoperative transfusion of 2 units of red blood cells.

## Discussion

Although the anti-C5 antibodies eculizumab and the long-lasting ravulizumab have been established as the standard of care for PNH patients, effectively reducing adverse events and mortality, unmet needs persist in cases of treatment failure [3,4]. Several novel drugs such as pegcetacoplan [5], danicopan [6], crovalimab [7], and iptacopan [1] have recently emerged, significantly changing the management of patients with PNH. Among these, Iptacopan is a first-in-class, oral, selective factor B inhibitor that targets the alternative complement pathway and has demonstrated substantial efficacy in controlling hemolysis and reducing transfusion burden in PNH patients [1]. Its oral administration offers advantages in convenience over intravenous or subcutaneous administrated drugs, but also raises concerns of adherence and rapid loss of complement control upon discontinuation due to its shorter half-life. Perioperative management of iptacopan remains unaddressed in the literature, and to our knowledge, this is the first reported case of successful elective surgery without BTH after short-term (two doses) iptacopan interruption in the perioperative period.

Perioperative management is a critical consideration in PNH because surgery is considered a complement amplifying condition that can trigger hemolysis and thrombosis in PNH patients. There are several case series that major surgeries were performed without complications under the treatment of eculizumab [8, 9, 10]. Today, several guidelines recommend giving a dose of eculizumab one day before surgery [11]. Kimura et al. [12] reported that following the eculizumab recommendation, ravulizumab administration one day before laparoscopic cholecystectomy led to an uneventful perioperative period in a PNH patient. In contrast, due to their recent approval and limited clinical experience, data regarding perioperative management with novel agents remain scarce. For pegcetacoplan, a subcutaneous anti-C3 antibody administered twice weekly, there is one perioperative case report available. Vara et al. [13] described successful perioperative management in a PNH patient undergoing multiple surgeries, achieved through dose adjustments of pegcetacoplan and careful monitoring of hemolytic markers. Further reports on the perioperative management of these agents are awaited; the accumulation of such data is expected to contribute to the development of clinical evidence and new guidelines. Therefore, our case supports the notion that a one-day iptacopan holiday, in the setting of scheduled surgery, may not compromise complement control in well-stabilized patients. In this patient, the decision to interrupt iptacopan for one day was based on its pharmacokinetic profile (T_max ≈ 2 h, half-life 18–25 h), the planned minimally invasive procedure, and the ability to resume oral intake promptly. Importantly, backup intravenous complement inhibition with eculizumab was arranged in case of prolonged oral intake restriction or evidence of BTH. Close perioperative laboratory monitoring allowed for timely detection of potential hemolysis, with only a mild transient LDH elevation that resolved spontaneously.

The main limitation of this observation is that it represents a single patient undergoing a laparoscopic procedure with an uncomplicated postoperative course. The safety of perioperative iptacopan interruption in higher-risk scenarios, such as major surgery, delayed resumption of oral intake, or active hemolysis, remains unknown. Even so, we present this case with the view that the accumulation of such individual reports will provide valuable evidence to permit the development of future clinical guidelines. Also, it highlights the importance of individualized planning, backup complement inhibition, and close monitoring. Given the expanding therapeutic landscape in PNH, with the introduction of multiple novel complement inhibitors, it is essential to develop perioperative management strategies tailored to each drug’s pharmacologic characteristics.

## Conclusion

This case demonstrates that a planned, short-term interruption (one day) of iptacopan during the perioperative period can be tolerated without breakthrough hemolysis in a stable PNH patient undergoing elective laparoscopic surgery. Careful preoperative planning, availability of backup intravenous complement inhibition, and close perioperative monitoring are essential. Further clinical data are needed to confirm the safety and define best practices for perioperative management of iptacopan and other newly approved complement inhibitors.

## Informed consent

Informed consent was obtained from the patient for publication of this case report.

## Author contributions

T.K. drafted the initial manuscript. J.O. provided critical revisions and edited the manuscript. H.T. supervised the project and finalized the manuscript. All authors were involved in the diagnosis, treatment, and follow-up of the patient, critically revised the manuscript, read, and approved the current version of the manuscript.

## Data availability statement

The data that support the findings of this study are available from the corresponding author upon reasonable request.

## Declaration of generative AI and AI-assisted technologies in the writing process

During the preparation of this work the authors used ChatGPT (OpenAI) in order to assist with English language editing during the manuscript preparation. After using this tool/service, the authors reviewed and edited the content as needed and take full responsibility for the content of the publication

## Conflicts of interest

RS received honoraria for lectures from Takeda, GSK, Janssen, BMS, Nippon Shinyaku, Sanofi, Ono Pharmaceutical, Chugai Pharmaceutical and Kyowa Kirin. HT received honoraria for lectures from the following companies: Kissei Pharmaceutical, Nippon Shinyaku, BMS, Towa Pharmaceutical, Kyowa Kirin, Chugai Pharmaceutical, PharmaEssentia, AbbVie, Alexion, Novartis, Nihon Kayaku, Janssen, Ono Pharmaceutical, Otsuka Pharmaceutical, Daiichi Sankyo, Takeda, Meiji Seika Pharma, Aska Pharmaceutical, Astellas Pharma, Genmab, Asahi Kasei, Gilead Sciences, Eisai, AstraZeneca.
